# RNA-sequencing data-driven dissection of human plasma cell differentiation reveals new potential transcription regulators

**DOI:** 10.1038/s41375-021-01234-0

**Published:** 2021-04-06

**Authors:** Alboukadel Kassambara, Laurie Herviou, Sara Ovejero, Michel Jourdan, Coraline Thibaut, Veronika Vikova, Philippe Pasero, Olivier Elemento, Jérôme Moreaux

**Affiliations:** 1 Department of Biological Hematology, CHU Montpellier, Montpellier, France; 2 IGH, CNRS, University of Montpellier, Montpellier, France; 3 Englander Institute for Precision Medicine, Institute for Computational Biomedicine, Weill Cornell Medical College, New York, NY USA; 4 University of Montpellier, UFR Medicine, Montpellier, France; 5 Institut Universitaire de France (IUF), Paris, France

**Keywords:** Plasma cells, Differentiation

## Abstract

Plasma cells (PCs) play an important role in the adaptive immune system through a continuous production of antibodies. We have demonstrated that PC differentiation can be modeled in vitro using complex multistep culture systems reproducing sequential differentiation process occurring in vivo. Here we present a comprehensive, temporal program of gene expression data encompassing human PC differentiation (PCD) using RNA sequencing (RNA-seq). Our results reveal 6374 differentially expressed genes classified into four temporal gene expression patterns. A stringent pathway enrichment analysis of these gene clusters highlights known pathways but also pathways largely unknown in PCD, including the heme biosynthesis and the glutathione conjugation pathways. Additionally, our analysis revealed numerous novel transcriptional networks with significant stage-specific overexpression and potential importance in PCD, including BATF2, BHLHA15/MIST1, EZH2, WHSC1/MMSET, and BLM. We have experimentally validated a potent role for BLM in regulating cell survival and proliferation during human PCD. Taken together, this RNA-seq analysis of PCD temporal stages helped identify coexpressed gene modules with associated up/downregulated transcription regulator genes that could represent major regulatory nodes for human PC maturation. These data constitute a unique resource of human PCD gene expression programs in support of future studies for understanding the underlying mechanisms that control PCD.

## Introduction

Representing the end stage of B-cell differentiation, plasma cells (PCs) play an important role in humoral immunity by synthesizing and secreting antibodies, thus protecting the host against infections [1]. We previously developed a multistep culture system with various combinations of cytokines and activation molecules that reproduce the sequential PC differentiation (PCD) occurring in the different organs/tissues in vivo [2–5]. PCD is initiated by activation of B cells, leading to their differentiation into transitional preplasmablasts (prePBs), a highly proliferating cell population [3]. These prePBs further differentiate into plasmablasts (PBs), which can develop into quiescent long-lived PCs after migrating to survival niches which are traditionally in the bone marrow [3, 6]. Specific pro-survival niches could also comprise mucosa and sites of inflammation [7]. B cells and PCs are key players of humoral immunity. Understanding the biological processes that control the production and the survival of normal PCs is critical both to prevent tumorigenesis and identify targets for pathogenic PCs and ensure efficient immune response without autoimmunity or immune deficiency.

On the transcriptional level, the differentiation of B cells into PCs is associated with substantial and coordinated changes in gene expression profiles [6]. These changes are tightly guided by two sets of stage-specific transcription factors (TFs) that repress each other including: (1) B-cell TFs (PAX5, BCL6, and BACH2) maintaining the B-cell fate and (2) PC TFs (IRF4, BLIMP1 and XBP1) that are required to extinguish B-cell genes and activate the antibody-secreting cell (ASC) program [6, 8].

Over the past decades, much progress has been made in understanding the physiological and transcriptional processes occurring during PCD [3, 4, 6, 9–12]. Knowledge of global gene expression patterns during PCD has been largely based on data obtained in mouse systems. Recent studies using RNA sequencing (RNA-seq) provide a more comprehensive view of transcriptional changes during murine PCD [13].

Most human PCD transcriptome studies have been carried out using microarray techniques [3, 4, 9, 10]. Given the limitation of microarray technology, high-throughput sequencing technology is needed to fully characterize the temporal gene expression program operating during human PCD. In vitro differentiation of human memory B cells (MBCs) into PCs has been demonstrated to be a powerful model of human PCD [3, 4, 11].

In this study, we used next-generation sequencing technology to generate a comprehensive transcriptome database encompassing human in vitro PCD. Analyses of differentially expressed genes during PCD revealed 6374 genes that we organized into clusters of coexpressed genes based on temporal expression patterns. The major temporal programs we identified were associated with key pathways consistent with PC biology, as well as novel pathways with potential importance in PCD. Additionally, our analysis revealed 449 transcriptional regulators correlated with these temporal programs of gene expression. Novel transcription regulators with consistent and marked overexpression during PCD include BATF2, BHLHA15/MIST1, EZH2, WHSC1/MMSET, and BLM. Furthermore, our analysis identified many epigenetic actors upregulated at prePB stage, a critical step where cell proliferation is high and where immunoglobulin (Ig) secretion starts. Finally, we have experimentally validated a role of BLM in regulating cell survival and proliferation in PCD. Taken together, this analysis thus identifies a discrete set of genes that function together to regulate PCD. These data and results provide a unique resource to decipher major gene networks involved in human PC development and ultimately will help provide fundamental insight into the mechanisms that control PCD.

## Materials and methods

### Cell populations and mRNA extraction

prePBs, PBs, and PCs were generated using a three-step in vitro model starting from peripheral blood MBCs as reported [3, 4]. Peripheral blood cells from healthy volunteers were purchased from the French Blood Center (Toulouse, France) and CD19^+^ CD27^+^ MBCs purified (>95% purity) as described [3]. Standard culture conditions comprised 21% O2, 5% CO_2_, and 37 °C. PCs were generated as reported [2, 3]. Cultures were performed in Iscove’s modified Dulbecco medium (Invitrogen) and 10% FCS. Purified peripheral blood MBCs (1.5 × 10^5^/ml) were activated for 4 days by CpG oligodeoxynucleotide and CD40 ligand (sCD40L)—10 µg/ml of phosphorothioate CpG oligodeoxynucleotide 2006 (Sigma), 50 ng/ml histidine tagged sCD40L, and anti-poly-histidine mAb (5 µg/ml), (R&D Systems)—with IL-2 (20 U/ml), IL-10 (50 ng/ml), and IL-15 (10 ng/ml) in six-well culture plates. PBs were generated by removing CpG oligonucleotides and sCD40L and changing the cytokine cocktail (IL-2, 20 U/ml, IL-6, 50 ng/ml, IL-10, 50 ng/ml, and IL-15, 10 ng/ml). PBs were differentiated into PCs adding IL-6 (50 ng/ml), IL-15 (10 ng/ml), and IFN-α (500 U/ml) for 3 days. PrePBs were purified at day 4, PBs at day 7, and PCs at day 10 using Facs Aria cell sorter (Becton Dickinson) (Supplementary Fig. S1). We performed three independent experiments starting from purified MBCs of three different healthy donors. RNA was isolated from cells with Qiagen RNeasy Micro or Mini Kits (Qiagen, Hilden, Germany) according to the manufacturer’s instructions.

### RNA sequencing and data analysis

The RNA-seq library preparation was done with 150 ng of input RNA using the Illumina TruSeq Stranded mRNA Library Prep Kit. Paired-end RNA-seq were performed with illumina NextSeq sequencing instrument (Helixio, Clermont-Ferrand, France). RNA-seq read pairs were mapped to the reference human GRCh37 genome using the STAR aligner [14]. All statistical analyses were performed with the statistics software R (version 3.2.3; available from: https://www.r-project.org) and R packages developed by BioConductor project (available from: https://www.bioconductor.org) [15]. The expression level of each gene was summarized and normalized using the DESeq2 R/Bioconductor package [16]. A summary of read mapping and quantification results can be found in Supplementary Fig. S2. The RNA-seq data are available in Gene Expression Omnibus under the accession number GSE148924. The raw gene-wise read counts are provided in Supplementary File 1. Differential expression analyses were performed using DESeq2 pipeline. *p* values were adjusted to control the global FDR across all comparisons with the default option of the DESeq2 package. Genes were filtered from downstream analysis if they did not have a log2 mean normalized count value of at least 6 in at least one group. Genes were considered differentially expressed if they had an adjusted *p* value < 0.05 and a fold change > 2. Heat maps of gene expression were generated using the ComplexHeatmap R/Bioconductor package. Pathway enrichment analyses were performed using the R package ReactomePA [17].

### Human myeloma cell lines (HMCLs)

XG HMCLs were obtained as previously described [18]. JJN3 was kindly provided by Dr. Van Riet (Brussels, Belgium), JIM3 by Dr. MacLennan (Birmingham, UK), and MM1S by Dr. S. Rosen (Chicago, USA). AMO-1, LP1, L363, U266, OPM2, and SKMM2 were purchased from DSMZ (Braunsweig, Germany) and RPMI8226 from ATTC (Rockville, MD, USA). All HMCLs derived in our laboratory were cultured in the presence of recombinant IL-6. HMCLs were authenticated according to their short tandem repeat profiling and their gene expression profiling using Affymetrix U133 plus 2.0 microarrays deposited in the ArrayExpress public database under accession numbers E-TABM-937 and E-TABM-1088 [18].

### Clinical samples and gene expression data

Affymetrix data of purified MMC from a cohort of 282 patients with MM included in the DutchBelgian/German HOVON65/GMMG-HAD trial were used (GSE19784) (HOVON65/GMMGHD4 cohort) [19]. The clinical characteristics of this cohort have been previously described [19].

### Myeloma cell growth assay

HMCLs were cultured for 4 days, in 96-well flat-bottom microtiter plates, in RPMI 1640 medium, 10% FCS, and 2 ng/ml IL-6 (control medium) in the presence of ML216 (Sigma-Aldrich, St Louis, MO). The number of metabolic-active cells was also determined using intracellular ATP quantitation. Cell growth was evaluated by quantifying intracellular ATP amount with a Cell Titer Glo Luminescent Assay (Promega, Madison, WI, USA) using a Centro LB 960 luminometer (Berthold Technologies, Bad Wildbad, Germany).

### Validating the implication of BLM in PCD

ML216 (Sigma-Aldrich, St Louis, MO), the inhibitor of BLM helicase activity (1 µM), was added at the beginning of each PCD transition step and its effect on cell count, viability and cycle, was analyzed at the end of the step. DMSO treated cells were used as control. Cell count and viability were assessed with the trypan blue dye exclusion test. Cell cycle were assessed using DAPI staining (Sigma-Aldrich) and cells in the S phase using incubation with bromodeoxyuridine (BrdU) for 1 h and labeling with an anti-BrdU antibody (APC BrdU flow kit, BD Biosciences, San Jose, CA, USA) according to the manufacturer’s instructions [20]. Apoptosis was assayed with PE-conjugated Annexin V labeling (Becton Dickinson, San Jose, CA, USA) and fluorescence was analyzed on a LSR Fortessa X20 flow cytometer (Becton Dickinson).

## Results

### RNA-seq profiling of in vitro human PC differentiation

To obtain a global transcriptomic map of human PCD, we performed RNA-seq analysis of four in vitro human PCD subpopulations: MBCs, prePBs, PBs, and PCs [3, 4]. Approximately 50 million read pairs were generated for each RNA sample. The number of mapped reads per sample is provided in Supplementary Fig. S2.

**Fig. 1:**
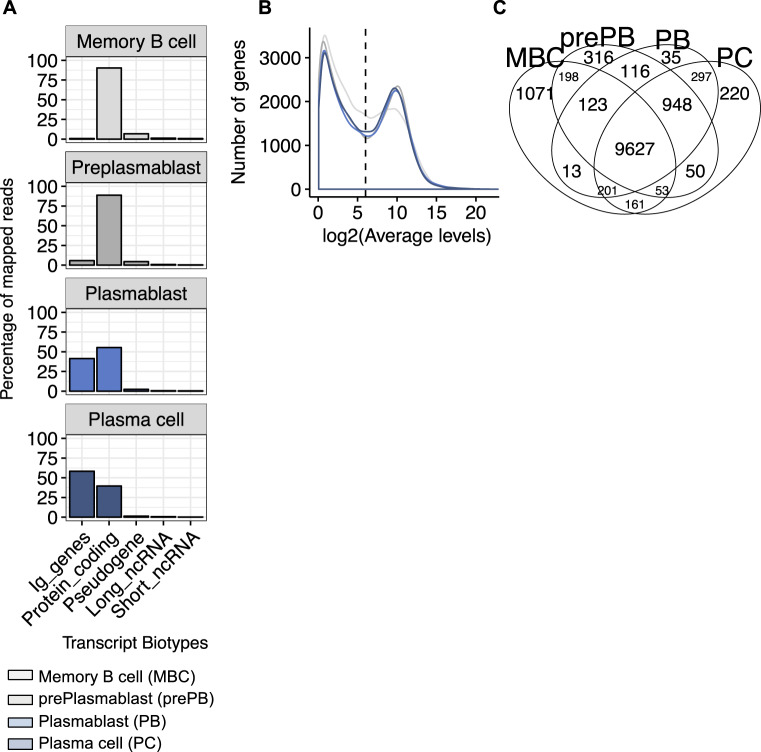
Transcriptome sequencing of human plasma cell differentiation. **A** The plot shows, for each cell subpopulation, the percentage of mapped reads per Ensembl transcript biotypes (protein-coding genes, pseudogene, long noncoding RNA, and short noncoding RNA). **B** Frequency distribution of the genes based on the average mRNA levels. Distribution of the average normalized gene read counts for each cell subpopulation is shown. The distribution is bimodal for all cell subpopulations, defining actively expressed genes with average normalized read counts ≥ 64. **C** Venn diagram of the numbers of expressed genes in MBC (memory B cell), prePB (preplasmablast), PB (plasmablast), and PC (plasma cell).

First, we determined the proportion of mapped reads per transcript classification in each cell subpopulation (Fig. 1A), based on Ensembl gene biotype annotation model. As expected, PCD is accompanied by a gradual increase of Ig gene expression. This increase starts from prePB stage and becomes more pronounced at PB and PC stages.

**Fig. 2:**
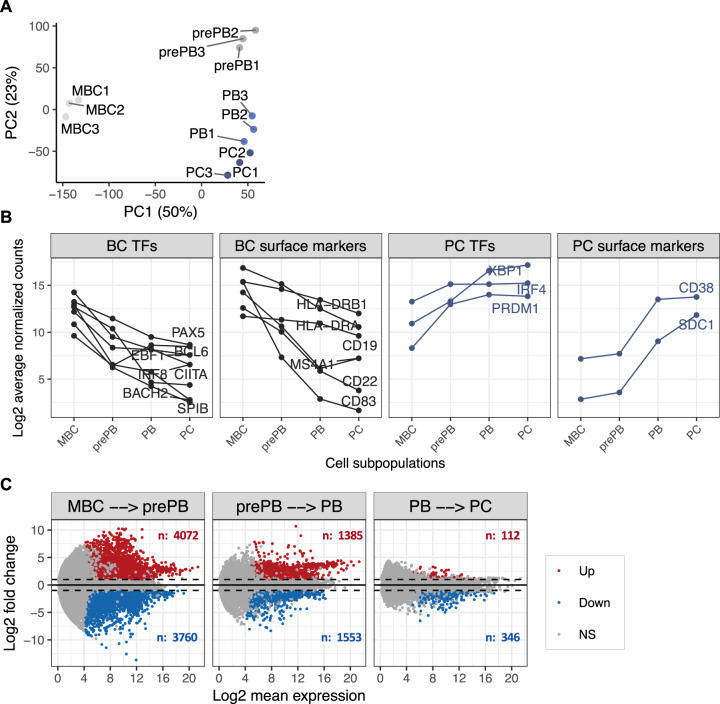
Differentially expressed gene signatures during human plasma cell differentiation. **A** Principal component analysis of genes expressed during PCD. **B** Expression profile of transcription factors and cell surface markers known in PCD. **C** MA plots of differentially expressed genes. Differentially expressed genes were identified using the DESeq2 R package (adjusted *p* value ≤ 0.05 and fold change ≥ 1.5). *p* values were adjusted using the BH algorithm multiple-testing correction. **A** MA (log ratio (M) versus mean average (A) expression) plot showing differentially expressed genes between two consecutive cell subpopulations. Significantly differentially upregulated genes are represented by red dots, while significantly differentially downregulated genes are represented by blue dots.

We wondered if the strong expression of Ig genes by PBs and PCs could restrict the expression profile of other genes. To evaluate this hypothesis, we estimated the total number of genes actively expressed in each cell subpopulation. We identified a normalized read count cutoff of 64 to define transcripts with active expression (Fig. 1B). Any gene with a mean DESeq2-normalized expression above 64, in at least one cell subpopulation, is defined as transcriptionally active. Using this criterion, 13,429 genes were classified as actively expressed in at least one B to PCD stage. Among them, 84–86% are expressed in each cell subpopulation, including PCs. This observation is consistent with previous reports by Shi et al. [13] in mouse model, indicating that, despite their strong functional specialization, ASC maintain a highly diverse gene expression repertoire similar to B cells. Among the 13,429 genes, 9627 genes (~71.7%) produced mRNAs detected in all cell subpopulations (Fig. 1C), whereas 3802 genes (28.3%) are expressed only during specific stages of PCD. Of these 3802 genes, 1071 genes are specifically detected in MBC, 316 genes in prePB, 35 in PB, 220 in PC, and 2160 in more than two specific stages (Fig. 1C). In an unsupervised principal component analysis of the 13,429 gene expression levels, B cell to plasma stages was segregated according to their developmental stage (Fig. 2A). Additionally, investigation of the expression profile for known genes involved in B cell to PCD, including *PAX5*, *BCL6*, *SPIB*, *BACH2*, *PRDM1*, *IRF4*, *CD38*, and *SDC1* confirmed the accuracy of our RNA-seq data (Fig. 2B).

We next focused our analysis on the dynamic expression changes during PCD by performing pairwise comparisons between two consecutive cell populations using the DESeq2 R package (adjusted *p* value ≤ 0.05, fold change ≥ 2) (Fig. 2C and Supplementary Table S1). Each gene was also required to have an average expression of ≥64 normalized count, in at least one of the two considered cell populations. Large numbers of genes are differentially expressed at different stages of human PCD. A list of 7832 genes was differentially expressed during MBC to prePB transition (up: 4072 genes, down: 3760 genes); 2938 genes during prePB to PB transition (up: 1385 genes, down: 1553 genes); 458 genes during PB to PC transition (up: 112 genes, down: 346 genes) (Supplementary Table S1). The differentiation stage showing the most pronounced transcriptome changes in comparison to the preceding one is prePBs (Fig. 2C). A total of 8890 unique genes are differentially expressed in one or more steps (Supplementary Fig. S3) during PCD, suggesting a complex dynamic transcriptome changes during the generation of PCs.

To better understand the gene expression changes occurring at each transition stage, we used the ReactomePA R/Bioconductor package to determine enriched molecular pathways (Supplementary Table S2). The top 20 significantly enriched pathways are shown for each PCD transition (Supplementary Fig. S4A, B). Consistent with known biology, the transition from MBC to prePB is mainly characterized by the activation of cell cycle pathways. The transition from prePB to PB is marked by a strong downregulation of cell cycle genes (Supplementary Fig. S4B). Overexpressed genes during prePBs to PBs transition are enriched in genes involved in protein production with increased metabolic activity (translation, modification, transport, unfolded protein response, and chaperones) (Supplementary Fig. S4A), underlying the increasing Ig-secreting capacity. The strongest change during PB to PC transition is mainly the downregulation of cell cycle genes (Supplementary Fig. S4B).

### Identification of temporal gene expression patterns during human PC differentiation

**Fig. 3:**
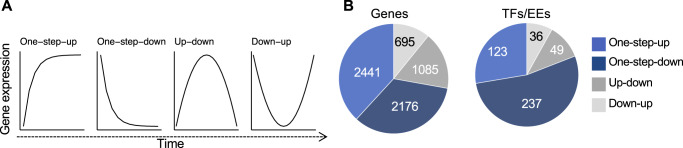
Stepprofiler package to extract temporal gene regulation during human plasma cell differentiation. **A** Basic temporal gene expression patterns. **B** Numbers of mRNAs and the coexpressed transcription factors (TFs) showing transitions from low to high (one-step-up) or high to low (one-step-down) in two consecutive differentiation stages and two-step-up-down (up-down) or two-step-down-up (down-up) in our series of differentiation stages.

Differentiation processes require expression changes and can therefore be accompanied by at least four general temporal gene expression patterns [21] that we refer to as one-step-up or one-step-down (mRNA level transitions from low to high or high to low, respectively, in two consecutive differentiation stages) and two-step-up-down or two-step-down-up (i.e., impulse-down) (mRNA level transitions from low to high and back down or from high to low and back up, respectively, in a series of differentiation stages) (Fig. 3A).

We built a R package named stepprofiler (https://github.com/kassambara/stepprofiler) to extract the temporal gene expression patterns of human PCD. This analysis identified 8419 genes (Supplementary Table S3) with one or two transition points in expression during PCD (Fig. 3 and Supplementary Fig. S5). About 58% of the identified genes (including 68% of TF genes) showed a single transition point; of these, 30% exhibited the one-step-up pattern, and 28% showed the one-step-down pattern. By contrast, 42% of genes exhibited two transition points (two-step-up-down and two-step-down-up genes) (Fig. 3B).

**Fig. 4:**
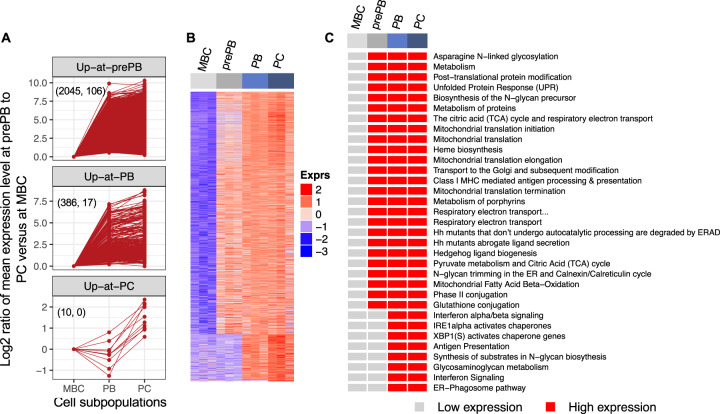
Temporal gene expression profiling during human plasma cell differentiation. **A** Identification of the one-step-up transition in mRNA levels for all genes and the coexpressed TFs. Three expression patterns were identified to represent genes up-regulated at prePB stage, at PB stage or at PC stage. Gene expression profiles of individual genes are depicted. The total number of all genes (left) and the coexpressed transcription factor genes (right) are indicated in parentheses for each expression pattern. **B** Heatmap showing the expression profile of one-step-up genes. **C** Pathways enriched in one-step-up genes.

We further classified the genes based on the differentiation stage associated with the major expression transition (Fig. 4A, B and Supplementary Fig. S5). For example, “up-at-prePB” genes showed lower expression at MBC step and higher expression during prePB—PC, and “up-at-PB” genes showed lower expression at MBC—prePB and higher expression during PB—PC (Supplementary Fig. S5). With genes showing a single transition point (i.e., one-step-up and one-step-down genes), the transition in expression level occurred more frequently at prePB step indicating that a major transcriptional reprogramming occurs during the prePB stage (Supplementary Fig. S5C).

A stringent Reactome pathway enrichment analysis of these unique expression patterns identified key pathways showing significant enrichment at two differentiation stages (Fig. 4C). Among the pathways significantly upregulated at prePB, protein modifications and metabolisms, unfolded protein response, citric acid (TCA) cycle and respiratory electron transport, mitochondrial translation, heme biosynthesis and metabolism of porphyrins and glutathione conjugation were identified (Fig. 4C and Supplementary Table S4). Pathways upregulated at PB include interferon alpha/beta signaling, IRE1alpha activates chaperones, XBP1(S) activates chaperone genes, synthesis of substrates in N-glycan biosynthesis, glycosaminoglycan metabolism, and endoplasmic reticulum (ER) phagosome pathway. These data indicate that gene expression programs in PCD are dramatically reorganized at prePBs in preparation for the onset storage protein and protein production (Fig. 4C). Various important biological processes—most notably cell cycle, RHO GTPases activate formins, and DNA repair—operate by expression in short impulse manner at prePB stage (Supplementary Fig. S6).

### Identification of transcription factor/epigenetic enzyme (EE) repertoires of human PC differentiation

**Fig. 5:**
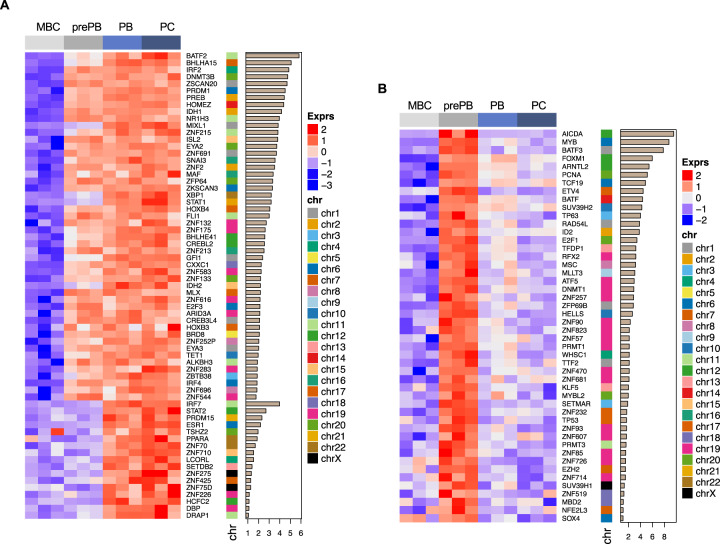
Transcription factors and/or epigenetic enzymes expressed during plasma cell differentiation. The heat maps show the relative expression profile (*z*-scores) of **A** one-step-up and **B** two-step-up-down TFs/EEs. Genes are sorted according to the maximum of fold change expression at each transition.

To better understand the nature of the regulatory processes involved in human PCD, we focused on TF and EE genes. We crossed our data with the 1391 census human sequence-specific DNA binding TFs [22] (Supplementary Table S5). Given the importance of EE genes in gene expression regulation, we also used a comprehensive list previously reported [23] (Supplementary Table S6). Collectively, we identified 445 TF/EE temporally regulated genes during PCD (Fig. 3B and Supplementary Table S7). One hundred and twenty-three TF/EE genes fall into the one-step-up groups (Figs. 3B and 5A). Among them, most are up-at-prePB (81%) groups (Fig. 3B and Supplementary Table S7). Two hundred thirty-seven TF/EE genes are included in one-step-down groups, 49 in the two-step-up-down groups, and 36 in the two-step-down-up groups (Fig. 3B and Supplementary Table S7). These results suggest that the specific patterns of gene expression detected during PCD are associated with specific TF/EE gene transcriptional changes.

We next focused on TFs/EEs exhibiting a one-step-up or two-step-up-down pattern (Fig. 5). We selected these groups because they are highly expressed in one or more PCD stages. Furthermore, genes associated with specific differentiation stages would be expected to be activated at specific time points during the differentiation progression. Among the one-step-up TFs/EEs are those encoding well-known PC TFs such as *IRF4, BLIMP1/PRDM1*, and *XBP1* (Fig. 5A). Interestingly, we found many other new TFs with less characterized function in PCD and a potential importance. New TFs with the most consistent and marked overexpression during B to PCD are *BATF2, BHLHA15, IRF2, ZSCAN20, MIXL1, MAF, ZKSCAN3*, and *STAT1* (Fig. 5A). Furthermore, our analysis identified EE genes consistently upregulated during PCD (Fig. 5A and Supplementary Table S7) including histone methyltransferases (PRDM1, PRDM15, PRMT7, SETDB2, SMYD2, and SMYD4), de-novo DNA methylation enzyme (DNMT3B), DNA methylation readers (MBD1 and ZBTB38), DNA methylation editors/erasers (IDH1, IDH2, TET1, ALKBH1, ALKBH3, and MGMT), and histone phosphorylation editor (EYA2 and EYA3). Additionally, our results reveal several interesting TFs/EEs genes among the genes included in the two-step-up-down group, including AICDA, MYB, BATF3, FOXM1, ARNTL2, SUV39H2, WHSC1, MYBL2, TP53, EZH2, and SUV39H1 that are specifically upregulated in the PrePB stage (Fig. 5B). This analysis thus identifies a discrete set of genes that may function together to program B-cell terminal differentiation. Many of these genes have not yet been described in PC biology. Together, the temporal RNA-seq analysis of PCD helped identify new TF/EE genes coexpressed with functional pathways that could represent major regulatory nodes involved in the control of PCD.

### Evaluating the role of BLM in PC differentiation

**Fig. 6:**
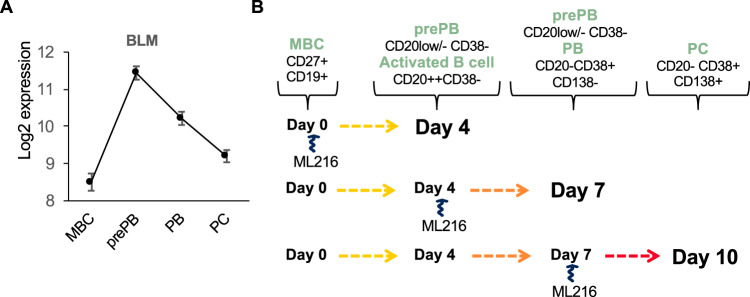
BLM is upregulated in preplasmablasts during plasma cell differentiation. **A** BLM gene expression profile in PCD cell subpopulations using RNA-seq. **B** Inhibition of BLM using the ML216 inhibitor (1 μM). The effect of BLM inhibition on each differentiation step was analyzed by adding the drug at the beginning of the differentiation step and analyzing its effect at the end.

**Fig. 7:**
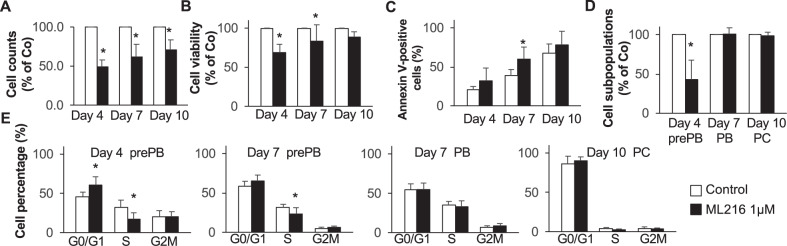
BLM inhibition affects human plasma cell differentiation. **A**, **B** Global cell counts and cell viability after treatment using trypan blue assay. Results are the mean absolute counts or viability ± SD of three independent experiments. **C** Analysis of apoptosis induction using Annexin V-PE staining by flow cytometry. The shown data are the mean values of three independent experiments. **D** Proportion of each cell subpopulation at the different time points of the plasma cell differentiation was determined by flow cytometry. **E** Analysis of cell cycle with flow cytometry using DAPI, BrdU incorporation, and labeling with an anti-BrdU antibody. The data are the mean values ± SD of three separate experiments.

prePBs are highly proliferative, express cytoplasmic Igs but not B cells or PC markers, and secrete Igs at a lower level than PBs or PCs. Accordingly, in this preplasmablastic stage, DNA replication and transcription need to be tightly coordinated to preserve the integrity of the genome of PrePBs. The RecQ family of DNA helicases is a family of conserved enzymes that display specialized and vital roles in the maintenance of genome stability [24, 25]. BLM, WRN, and RECQL4 are associated with genetic disorders characterized by chromosomal instability, premature aging, and increased susceptibility to cancer [24]. Patients with Bloom syndrome also suffer from recurrent infection that has suggested deficient immune function, even though these defects are less severe than in primary immunodeficiencies [26]. As previously reported for RECQ1 helicase [25], we also identified that high expression of *BLM* is associated with a poor outcome in newly diagnosed MM patients treated by high-dose therapy (HDT) and autologous stem cell transplantation (ASCT) (*p* = 0.003) (Supplementary Fig. S7A). Furthermore, high *BLM* expression is significantly associated with resistance to lenalidomide and SAHA HDACi in a large panel of human MM cell lines (*p* < 0.01 and *p* < 0.02, respectively) (Supplementary Fig. S7B, C). BLM inhibition using ML216 induced a significant toxicity in a panel of six different HMCLs with three sensitive cell lines and one more resistant (Supplementary Fig. S7D, E). Interestingly, BLM was identified as belonging to the two-step-up-down genes in association with cell cycle deregulation in prePBs (Fig. 6A). To investigate the role of BLM in PCD, we used a selective inhibitor (ML216) [27] of BLM’s helicase activity. We analyzed the effect of BLM inhibition on each differentiation step by adding 1 μM of ML216 at days 0, 4, and 7 (Fig. 6B). Inhibition of BLM activity resulted in a significant decrease of global cell count at days 4, 7, and 10 (Fig. 7A). Consistent with this result, we observed a significant decrease of cell viability at days 4 and 10 (Fig. 7B). The analysis of Annexin V-positive cells reveals a significant increase in apoptosis at day 7 (Fig. 7C). A close analysis showed that the BLM inhibition affects mainly the preplasmablastic stage characterized by BLM overexpression (Fig. 7D). Furthermore, ML216 treatment induced a cell cycle arrest of prePBs at days 4 and 7, with a significant inhibition of BrdU incorporation and an accumulation in the *G*_0_/*G*_1_ phase (*p* < 0.05) (Fig. 7E). Taken together, these data show that the inhibition of BLM affects the generation of prePBs leading to a significant defect in PCD.

## Discussion

Human normal PCs and their precursors are very difficult to obtain, as they are rare cells located in specific niches within the bone marrow and mucosa, hindering the understanding of their physiology and pathophysiology [28]. Consequently, insights into the molecular determinants of human PCD are generally inferred from nonhuman model system. We have developed and phenotypically characterized an in vitro model of PCD recapitulating the molecular characteristics of human PCD [2–4, 29].

Here, we investigated the dynamic transcriptional processes underlying human PCD using RNA-seq. We identified 6374 significantly differentially expressed genes classified into four temporal patterns (Figs. 3 and 4 and Supplementary Fig. S5). The temporal patterns fall into four major groups that we refer to one-step-up or one-step-down (mRNA level transitions from low to high or high to low, respectively, in two consecutive differentiation stages) and two-step-up-down (i.e., two-step-up-down) or two-step-down-up (i.e., impulse-down) (mRNA level transitions from low to high and back down or from high to low and back up, respectively, in a series of differentiation stages). The majority of the differentially expressed genes (72%) show a one-step-up or one-step-down pattern (Fig. 2B). Furthermore, most of the one-step-up and one-step-down genes exhibit an expression transition at the prePB stage (Fig. 2D and Supplementary Fig. S5B), suggesting that a dramatic reprogramming of the PCD transcriptome occurs at prePBs stage.

We found that genes upregulated during PCD were mainly involved in protein posttranslational modification, folding, trafficking, and metabolism (Fig. 2E). This is consistent with the known biology underlying the huge production of antibodies by PCs. Additionally, a stringent pathway enrichment analysis of these expression pattern highlights pathways largely unknown in PCD, including the heme biosynthesis and the glutathione conjugation pathways (Fig. 2E). The heme has been shown to directly bind and inhibit BACH2 function, resulting in the enhancement of the transcription of BLIMP1, the master regulator of PCs [30, 31]. Another interesting finding was the strong association between human PCD and the high expression of genes coding for proteins involved in mitochondrial functions and glutathione conjugation. During their differentiation to antibody-secreting PCs, B lymphocytes undergo dramatic changes in metabolism, structure, and function [32]. This transition entails extensive intracellular and extracellular redox changes, such as increased production of reactive oxygen species, followed by a strong antioxidant response [32]. Further studies elucidating the entire picture of heme pathway functions in PCD will provide valuable information for our understanding of the normal PC biology.

Furthermore, our data largely confirmed the specific expression patterns of known drivers of PC cell fate (IRF4, BLIMP1/PRDM1, and XBP1), but they also identified a number of other novel transcriptional regulators with potential importance in PCD including BATF2 and MIST1/BHLHA15.

BATF2 belongs to the AP-1/ATF superfamily of TFs. Recent studies have uncovered positive transcriptional activities of BATF family members in B cells, T cells, and dendritic cell [33]. BATF family members have been also described to interact with IRF4, a key PC TF [34–36], suggesting that BATF2 might be a key component of human PCD.

The TF MIST1/BHLHA15 has been recently described as a marker of murine and human PCs [37]. However, its role in PCD remains to be investigated. MIST1 has been recently identified as a “scaling factor” necessary to induce and maintain secretory cell architecture [38]. In gastric zymogenic cells, MIST1 expression is activated by XBP1, which also induces the expansion of the rough ER necessary to generate the massive loads of protein cargo to be packaged into the large, MIST1-mediated granules [39, 40]. The TF XBP1 plays a central role in regulating the UPR gene expression program [41], and as a consequence, is essential for the secretion of Igs by PCs [41, 42]. Recent studies revealed that MIST1 functions as a feedback regulator of the XBP1 gene [43]. These results suggest that MIST1/BHLHA15 might be critical in PCs and might co-operate with XBP1 to regulate a complex network of genes involved in antibody secretory function, as well as, in ER stress control.

Importantly, our results revealed several potential interesting TFs and epigenetic modifiers with a short impulse expression profile only at preplasmablastic stage (Fig. 3B). The prePBs are characterized by active proliferation and the beginning of Ig secretion. At this stage, proliferation, DNA replication, and transcription need to be tightly regulated. Consistently, among the genes upregulated at the prePB stage, we have identified many epigenetic actors, including histone methyltransferases (WHSC1/MMSET, EZH2), protein arginine methyltransferases (PRMT1 and PRMT3), DNA methylation enzyme (DNMT1), and DNA methylation reader (MBD2) (Fig. 3B). Among them, EZH2 was reported recently to play a key role during B to PCD supporting the maintenance of transitory immature proliferative state to support prePB amplification before differentiation [2]. Accordingly, EZH2 inhibition results in B to PC transcriptional changes together with induction of PC maturation and higher Ig secretion [2]. Many additional important genes were also upregulated, including genes involved in cell cycle, DNA replication, DNA repair, as well as, in DNA unwinding, such as members of RECQ family helicases (BLM and RECQ1) (Supplementary Fig. S6). Interestingly, we have recently shown that RECQ1 promotes resistance to replication stress and genotoxic agents in malignant PCs [25].

Here, we show that the inhibition of BLM affects the generation of prePBs leading to a decrease in PCD. BLM overexpression at the preplasmablastic stage may be important to support the replicative stress characterizing this differentiation step. However, the mechanism by which BLM executes this function in prePBs is currently unclear. A large body of evidence indicates that BLM plays a central role in the repair of stalled replication forks [44, 45]. BLM has also been implicated in the resolution of G-quadruplexes and of cotranscriptional R-loops, which form at highly expressed genes and represent a major source of replication impediments [46–48]. Moreover, recent evidence indicates that BLM connects DNA damage to the innate immune response and plays an important function in restraining unscheduled ISG induction under replication stress conditions [49]. It is therefore tempting to speculate that BLM could be important to restrain the deleterious consequences of replication–transcription conflicts in highly proliferative prePB cells upon activation of novel transcription programs. In the absence of BLM, these cells could accumulate stalled forks and chromosome breaks due to their inability to remove R-loops and G4 structures and to repair arrested forks. They would also trigger a type I interferon response, which would interfere with their normal differentiation process and together with the persistence of chromosome breaks, could contribute to cancer development.

It was demonstrated that, upon proteasome inhibitor treatment, clonal malignant prePBs can be detected in patients with Multiple Myeloma [50]. The prePBs lack full secretory status and produce less Igs, and thus are less sensitive to the ER stress induced by proteasome inhibitors [50, 51]. In this context, BLM inhibition could represent a therapeutic interest to target malignant prePBs involved in resistance to proteasome inhibitors in MM [50]. Furthermore, we identified that high *BLM* expression is associated with a poor outcome in newly diagnosed multiple myeloma patients treated by HDT and ASCT and with resistance to lenalidomide and HDACi. BLM inhibition could also represent a therapeutic strategy to target high-risk MM patients characterized by high BLM expression.

Altogether, the RNA-seq analysis of temporal stages of PCD helped to identify coexpressed gene sets with associated up/downregulated TF/EE genes that could represent important regulatory nodes of PCD. The nature of the relationships between the various genes and their regulatory factors remains to be determined. These data thus provide critical insights into new transcriptional events that sustain PC cell fate and differentiation.

## Supplementary information

Supplementary Figures

Supplementary Figure legends

Supplementary file 1

Supplementary Table S1

Supplementary Table S2

Supplementary Table S3

Supplementary Table S4

Supplementary Table S5

Supplementary Table S6

Supplementary Table S7

## References

[1] Shapiro-Shelef M, Calame K (2005). Regulation of plasma-cell development. Nat Rev Immunol.

[2] Herviou L, Jourdan M, Martinez AM, Cavalli G, Moreaux J (2019). EZH2 is overexpressed in transitional preplasmablasts and is involved in human plasma cell differentiation. Leukemia.

[3] Jourdan M, Caraux A, Caron G, Robert N, Fiol G, Reme T (2011). Characterization of a transitional preplasmablast population in the process of human B cell to plasma cell differentiation. J Immunol.

[4] Jourdan M, Caraux A, De Vos J, Fiol G, Larroque M, Cognot C (2009). An in vitro model of differentiation of memory B cells into plasmablasts and plasma cells including detailed phenotypic and molecular characterization. Blood.

[5] Jourdan M, Cren M, Robert N, Bollore K, Fest T, Duperray C (2014). IL-6 supports the generation of human long-lived plasma cells in combination with either APRIL or stromal cell-soluble factors. Leukemia.

[6] Nutt SL, Hodgkin PD, Tarlinton DM, Corcoran LM (2015). The generation of antibody-secreting plasma cells. Nat Rev Immunol.

[7] Lightman SM, Utley A, Lee KP (2019). Survival of long-lived plasma cells (LLPC): piecing together the puzzle. Front Immunol.

[8] Shaffer AL, Lin KI, Kuo TC, Yu X, Hurt EM, Rosenwald A (2002). Blimp-1 orchestrates plasma cell differentiation by extinguishing the mature B cell gene expression program. Immunity.

[9] Zhan F, Tian E, Bumm K, Smith R, Barlogie B, Shaughnessy J (2003). Gene expression profiling of human plasma cell differentiation and classification of multiple myeloma based on similarities to distinct stages of late-stage B-cell development. Blood.

[10] Tarte K, Zhan F, De Vos J, Klein B, Shaughnessy J (2003). Gene expression profiling of plasma cells and plasmablasts: toward a better understanding of the late stages of B-cell differentiation. Blood.

[11] Kassambara A, Jourdan M, Bruyer A, Robert N, Pantesco V, Elemento O (2017). Global miRNA expression analysis identifies novel key regulators of plasma cell differentiation and malignant plasma cell. Nucleic Acids Res.

[12] Caron G, Hussein M, Kulis M, Delaloy C, Chatonnet F, Pignarre A (2015). Cell-cycle-dependent reconfiguration of the DNA methylome during terminal differentiation of human B cells into plasma cells. Cell Rep.

[13] Shi W, Liao Y, Willis SN, Taubenheim N, Inouye M, Tarlinton DM (2015). Transcriptional profiling of mouse B cell terminal differentiation defines a signature for antibody-secreting plasma cells. Nat Immunol.

[14] Dobin A, Davis CA, Schlesinger F, Drenkow J, Zaleski C, Jha S (2013). STAR: ultrafast universal RNA-seq aligner. Bioinformatics.

[15] Gentleman RC, Carey VJ, Bates DM, Bolstad B, Dettling M, Dudoit S (2004). Bioconductor: open software development for computational biology and bioinformatics. Genome Biol.

[16] Love MI, Huber W, Anders S (2014). Moderated estimation of fold change and dispersion for RNA-seq data with DESeq2. Genome Biol.

[17] Yu G, He QY (2016). ReactomePA: an R/Bioconductor package for reactome pathway analysis and visualization. Mol Biosyst.

[18] Moreaux J, Klein B, Bataille R, Descamps G, Maiga S, Hose D (2011). A high-risk signature for patients with multiple myeloma established from the molecular classification of human myeloma cell lines. Haematologica.

[19] Kuiper R, Broyl A, de Knegt Y, van Vliet MH, van Beers EH, van der Holt B (2012). A gene expression signature for high-risk multiple myeloma. Leukemia.

[20] Requirand G, Robert N, Boireau S, Vincent L, Seckinger A, Bouhya S (2019). BrdU incorporation in multiparameter flow cytometry: a new cell cycle assessment approach in multiple myeloma. Cytometry B Clin Cytom.

[21] Bar-Joseph Z, Gitter A, Simon I (2012). Studying and modelling dynamic biological processes using time-series gene expression data. Nat Rev Genet.

[22] Vaquerizas JM, Kummerfeld SK, Teichmann SA, Luscombe NM (2009). A census of human transcription factors: function, expression and evolution. Nat Rev Genet.

[23] Yang Z, Jones A, Widschwendter M, Teschendorff AE (2015). An integrative pan-cancer-wide analysis of epigenetic enzymes reveals universal patterns of epigenomic deregulation in cancer. Genome Biol.

[24] Chu WK, Hickson ID (2009). RecQ helicases: multifunctional genome caretakers. Nat Rev Cancer.

[25] Viziteu E, Klein B, Basbous J, Lin YL, Hirtz C, Gourzones C (2017). RECQ1 helicase is involved in replication stress survival and drug resistance in multiple myeloma. Leukemia.

[26] Cunniff C, Bassetti JA, Ellis NA (2017). Bloom’s syndrome: clinical spectrum, molecular pathogenesis, and cancer predisposition. Mol Syndromol.

[27] Nguyen GH, Dexheimer TS, Rosenthal AS, Chu WK, Singh DK, Mosedale G (2013). A small molecule inhibitor of the BLM helicase modulates chromosome stability in human cells. Chem Biol.

[28] Radbruch A, Muehlinghaus G, Luger EO, Inamine A, Smith KG, Dorner T (2006). Competence and competition: the challenge of becoming a long-lived plasma cell. Nat Rev Immunol.

[29] Cocco M, Stephenson S, Care MA, Newton D, Barnes NA, Davison A (2012). In vitro generation of long-lived human plasma cells. J Immunol.

[30] Watanabe-Matsui M, Muto A, Matsui T, Itoh-Nakadai A, Nakajima O, Murayama K (2011). Heme regulates B-cell differentiation, antibody class switch, and heme oxygenase-1 expression in B cells as a ligand of Bach2. Blood.

[31] Jang KJ, Mano H, Aoki K, Hayashi T, Muto A, Nambu Y (2015). Mitochondrial function provides instructive signals for activation-induced B-cell fates. Nat Commun.

[32] Vene R, Delfino L, Castellani P, Balza E, Bertolotti M, Sitia R (2010). Redox remodeling allows and controls B-cell activation and differentiation. Antioxid Redox Signal.

[33] Murphy TL, Tussiwand R, Murphy KM (2013). Specificity through cooperation: BATF-IRF interactions control immune-regulatory networks. Nat Rev Immunol.

[34] Li P, Spolski R, Liao W, Wang L, Murphy TL, Murphy KM (2012). BATF-JUN is critical for IRF4-mediated transcription in T cells. Nature.

[35] Grusdat M, McIlwain DR, Xu HC, Pozdeev VI, Knievel J, Crome SQ (2014). IRF4 and BATF are critical for CD8(+) T-cell function following infection with LCMV. Cell Death Differ.

[36] Care MA, Cocco M, Laye JP, Barnes N, Huang Y, Wang M (2014). SPIB and BATF provide alternate determinants of IRF4 occupancy in diffuse large B-cell lymphoma linked to disease heterogeneity. Nucleic Acids Res.

[37] Capoccia BJ, Lennerz JK, Bredemeyer AJ, Klco JM, Frater JL, Mills JC (2011). Transcription factor MIST1 in terminal differentiation of mouse and human plasma cells. Physiol Genomics.

[38] Lo HG, Jin RU, Sibbel G, Liu D, Karki A, Joens MS (2017). A single transcription factor is sufficient to induce and maintain secretory cell architecture. Genes Dev.

[39] Huh WJ, Esen E, Geahlen JH, Bredemeyer AJ, Lee AH, Shi G (2010). XBP1 controls maturation of gastric zymogenic cells by induction of MIST1 and expansion of the rough endoplasmic reticulum. Gastroenterology.

[40] Metzler MA, Venkatesh SG, Lakshmanan J, Carenbauer AL, Perez SM, Andres SA (2015). A systems biology approach identifies a regulatory network in parotid acinar cell terminal differentiation. PLoS ONE.

[41] Shaffer AL, Shapiro-Shelef M, Iwakoshi NN, Lee AH, Qian SB, Zhao H (2004). XBP1, downstream of Blimp-1, expands the secretory apparatus and other organelles, and increases protein synthesis in plasma cell differentiation. Immunity.

[42] Reimold AM, Iwakoshi NN, Manis J, Vallabhajosyula P, Szomolanyi-Tsuda E, Gravallese EM (2001). Plasma cell differentiation requires the transcription factor XBP-1. Nature.

[43] Hess DA, Strelau KM, Karki A, Jiang M, Azevedo-Pouly AC, Lee AH (2016). MIST1 links secretion and stress as both target and regulator of the unfolded protein response. Mol Cell Biol.

[44] Urban V, Dobrovolna J, Janscak P (2017). Distinct functions of human RecQ helicases during DNA replication. Biophys Chem.

[45] Liao H, Ji F, Helleday T, Ying S. Mechanisms for stalled replication fork stabilization: new targets for synthetic lethality strategies in cancer treatments. EMBO Rep. 2018;19:e46263. 10.15252/embr.201846263 PMC6123652 30108055

[46] Garcia-Muse T, Aguilera AR (2019). Loops: from physiological to pathological roles. Cell.

[47] Chang EY, Novoa CA, Aristizabal MJ, Coulombe Y, Segovia R, Chaturvedi R (2017). RECQ-like helicases Sgs1 and BLM regulate R-loop-associated genome instability. J Cell Biol.

[48] Tan J, Duan M, Yadav T, Phoon L, Wang X, Zhang JM (2020). An R-loop-initiated CSB-RAD52-POLD3 pathway suppresses ROS-induced telomeric DNA breaks. Nucleic Acids Res.

[49] Gratia M, Rodero MP, Conrad C, Bou Samra E, Maurin M, Rice GI (2019). Bloom syndrome protein restrains innate immune sensing of micronuclei by cGAS. J Exp Med.

[50] Leung-Hagesteijn C, Erdmann N, Cheung G, Keats JJ, Stewart AK, Reece DE (2013). Xbp1s-negative tumor B cells and pre-plasmablasts mediate therapeutic proteasome inhibitor resistance in multiple myeloma. Cancer Cell.

[51] Orlowski RZ (2013). Why proteasome inhibitors cannot ERADicate multiple myeloma. Cancer Cell.

