# Supplementary material for: RNA-sequencing data-driven dissection of human plasma cell differentiation reveals new potential transcription regulators
Source: Leukemia. 2021 Apr 6;35(5):1451–62. doi: 10.1038/s41375-021-01234-0 (PMC8102200; doi:10.1038/s41375-021-01234-0)
Supplement: Supplementary file 1 — Supplementary Figures [file 41375_2021_1234_MOESM1_ESM.pdf]

Supplementary Figure S1

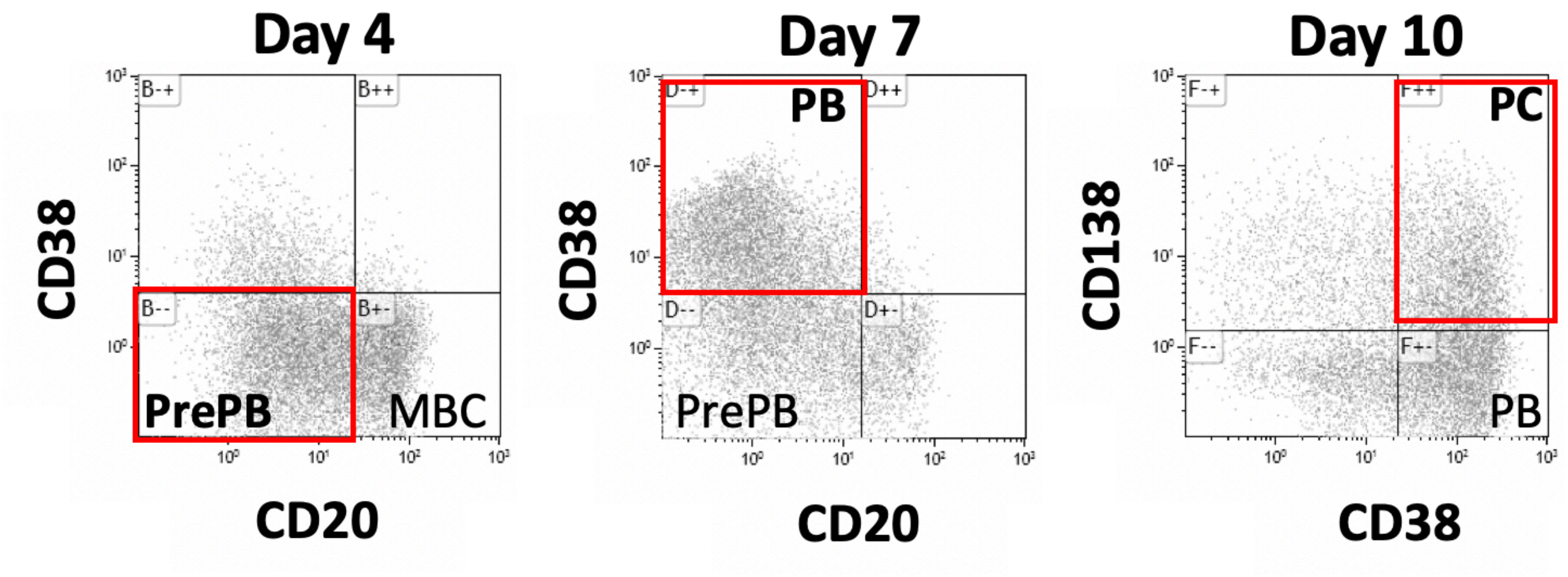

Supplementary Figure S2

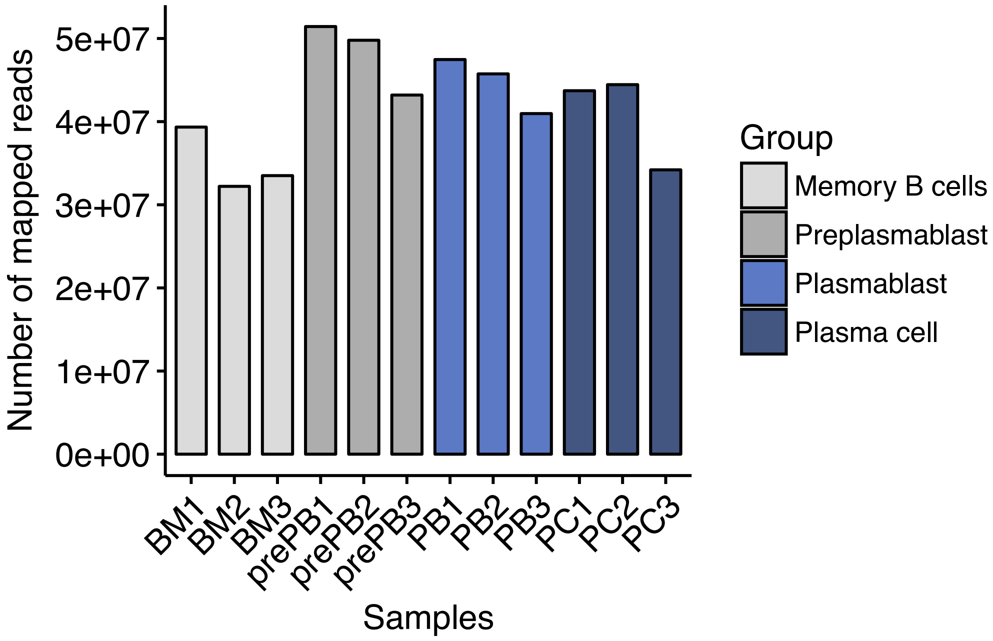

Supplementary Figure S3

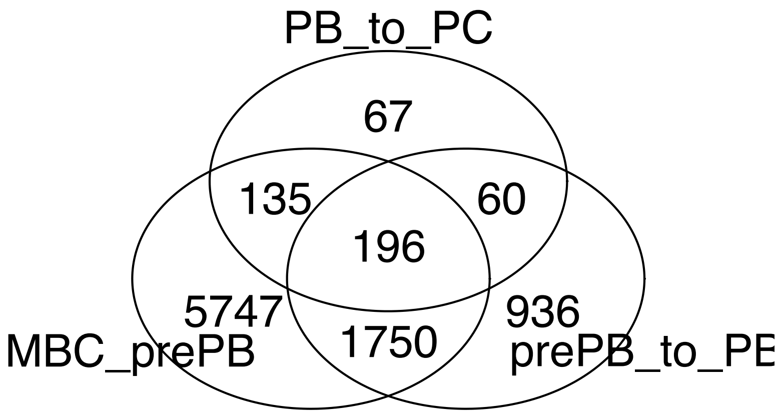

Supplementary Figure S4

A)

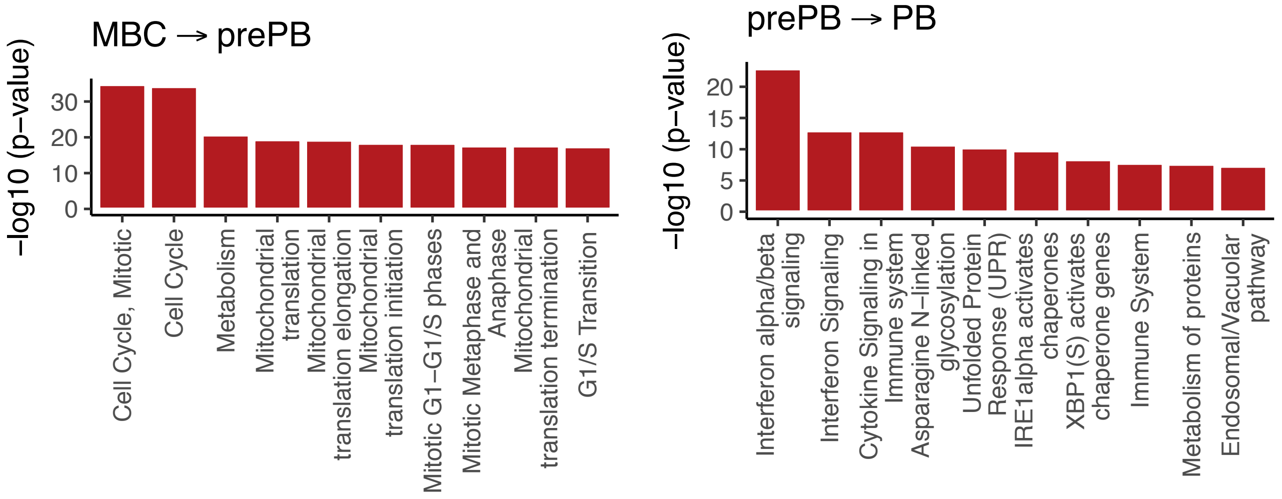

B)

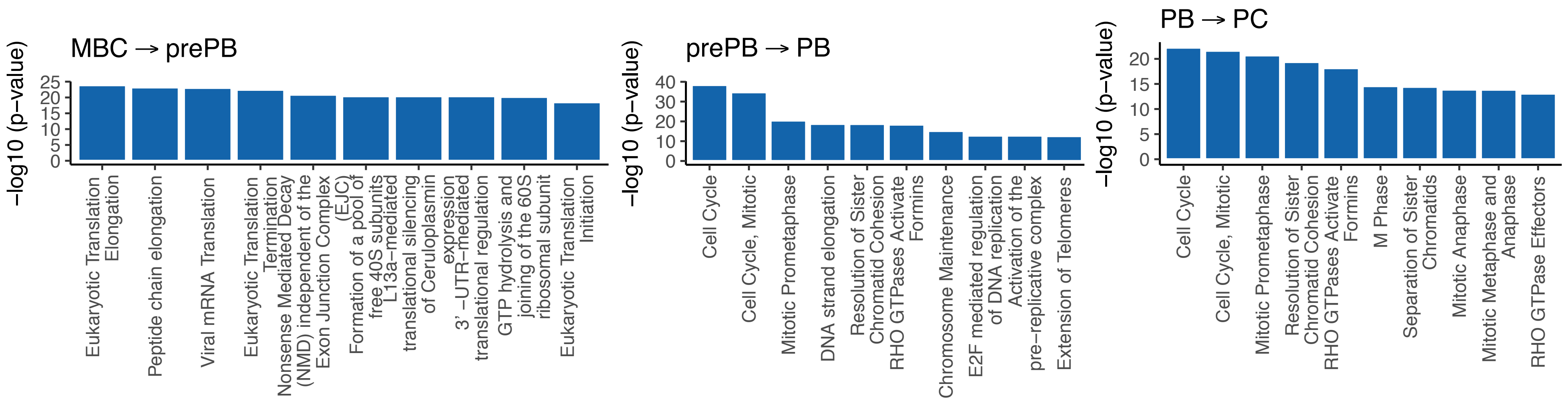

Supplementary Figure S5

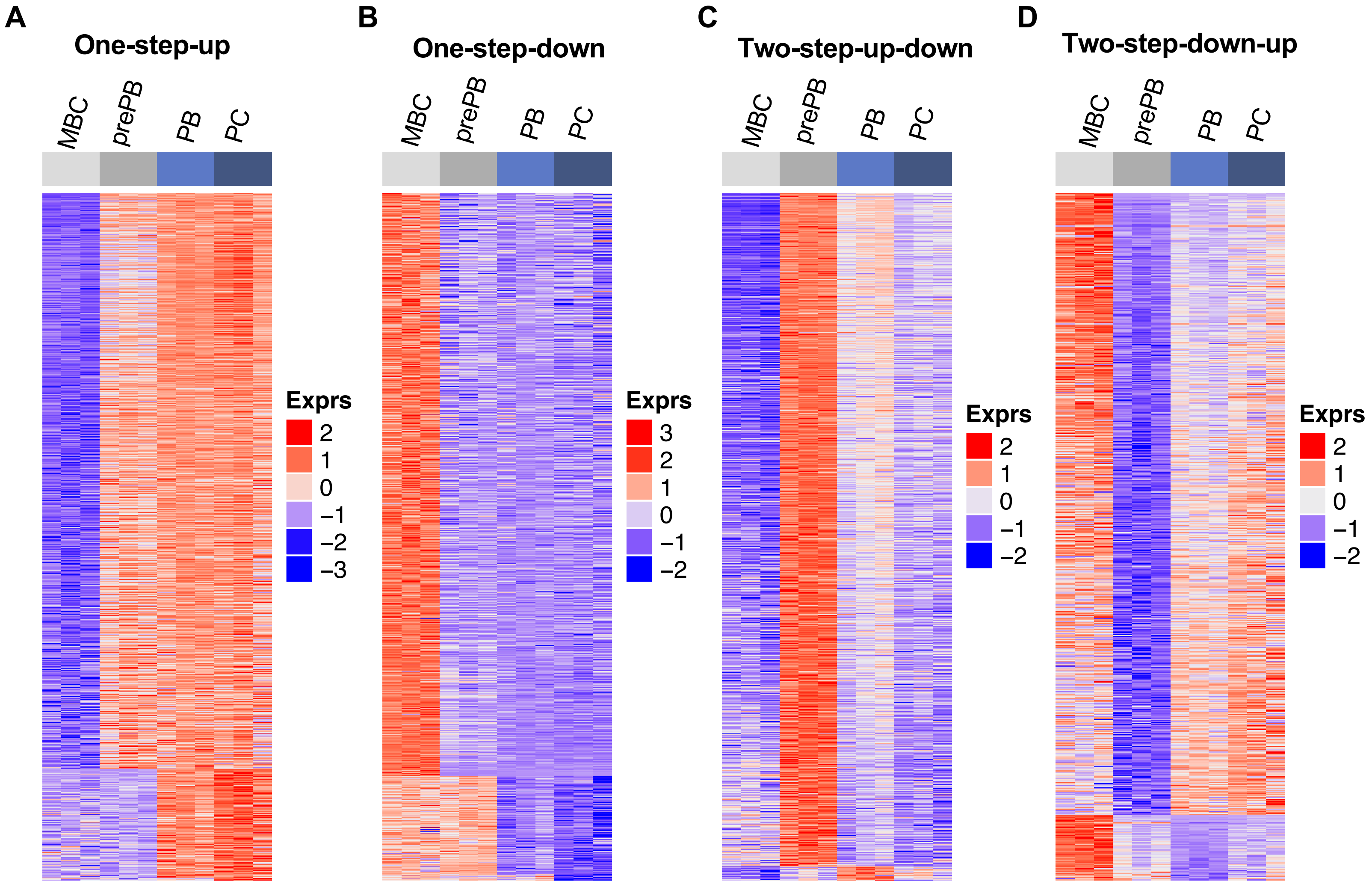

Supplementary Figure S6

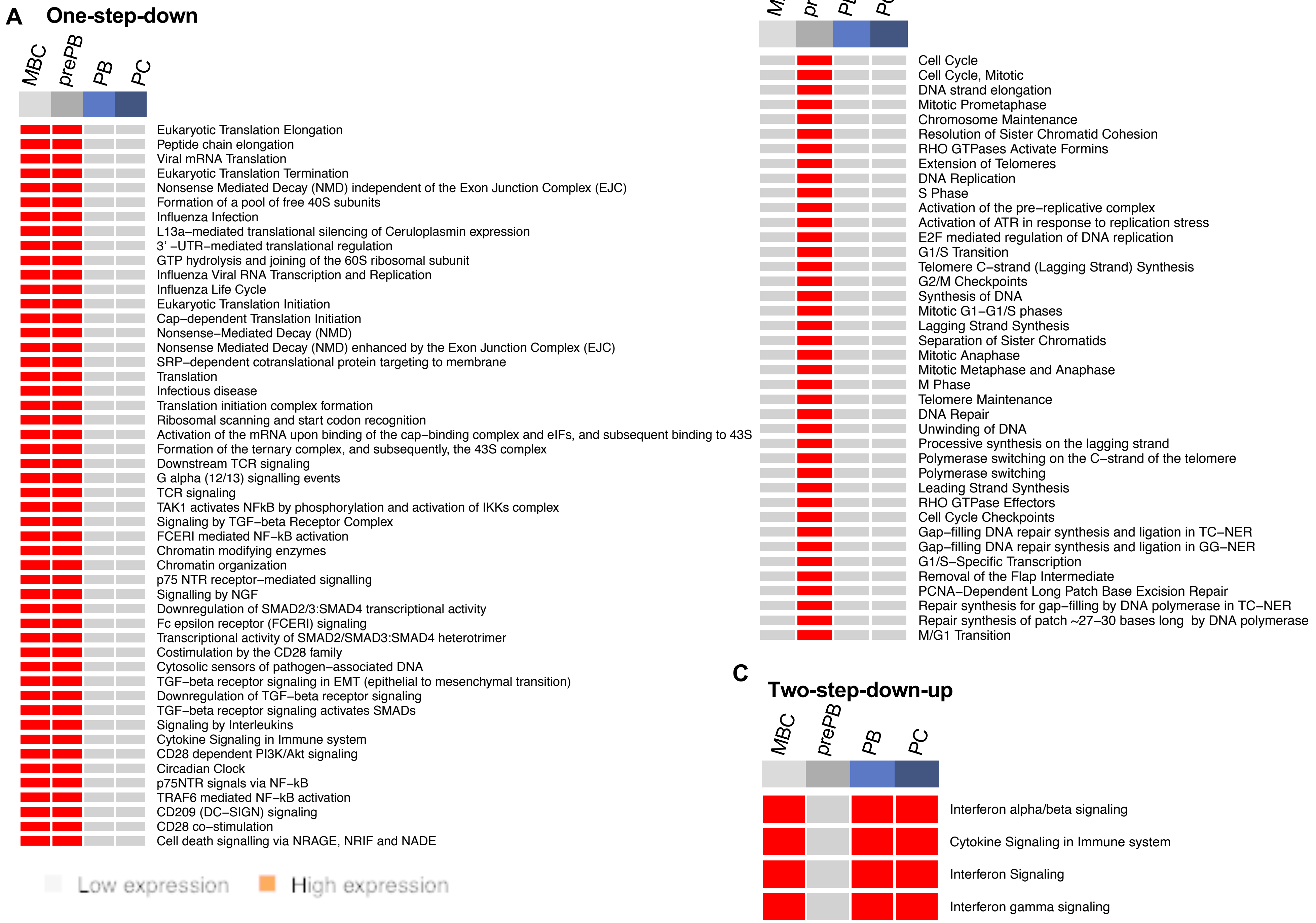

Supplementary Figure S7

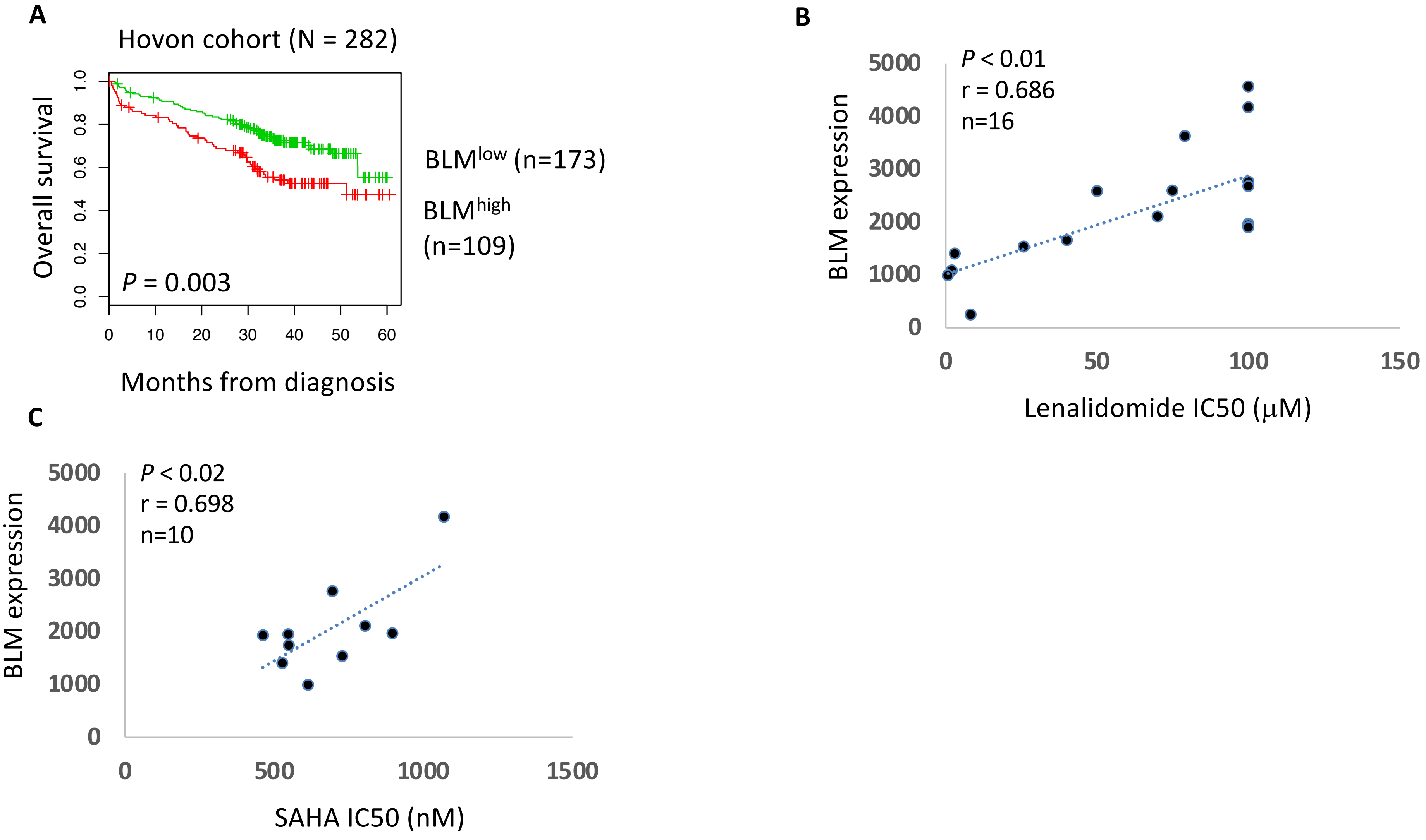

D

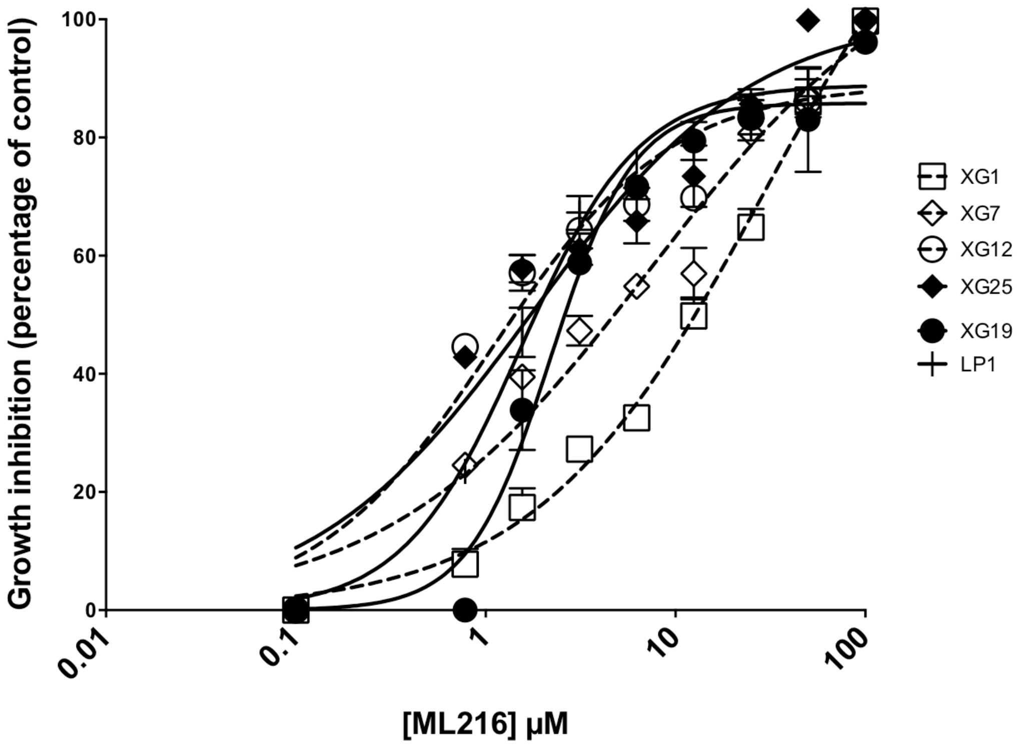

Growth inhibition (percentage of control)
100
80
60
40
20
0
0.01
0.1
1
10
100
[ML216] µM
XG1
XG7
XG12
XG25
XG19
LP1

E

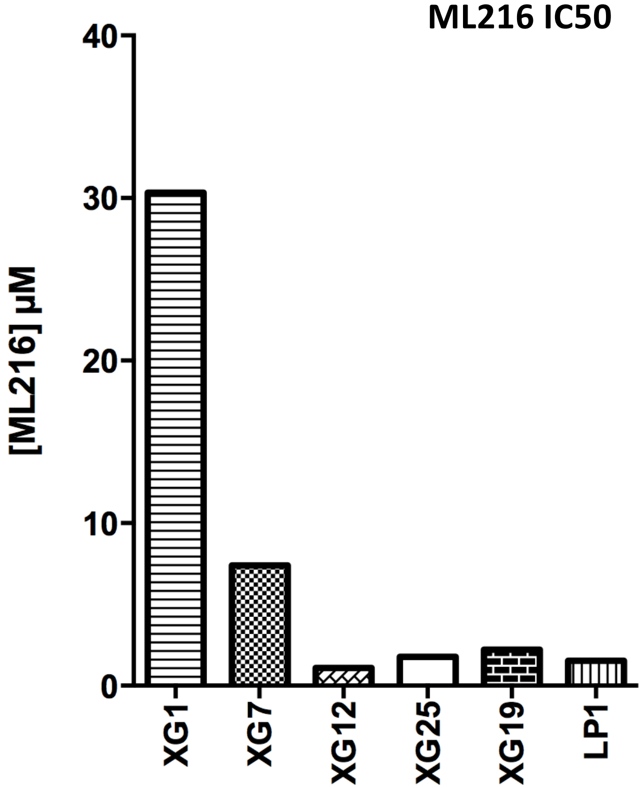

ML216 IC50
[ML216] µM
40
30
20
10
0
XG1
XG7
XG12
XG25
XG19
LP1
