# Supplementary material for: RNA-sequencing data-driven dissection of human plasma cell differentiation reveals new potential transcription regulators
Source: Leukemia. 2021 Apr 6;35(5):1451–62. doi: 10.1038/s41375-021-01234-0 (PMC8102200; doi:10.1038/s41375-021-01234-0)
Supplement: Supplementary file 2 — Supplementary Figure legends [file 41375_2021_1234_MOESM2_ESM.docx]

**Supplementary figure legends**

**Supplementary Figure S1**. In our in vitro model of B to PC differentiation, PrePBs were purified at day 4, PBs at day 7 and PCs at day 10 using Facs Aria cell sorter (Becton Dickinson). Red color indicates the gating strategy used for purification.

**Supplementary Figure S2**. Number of mapped read counts per sample using the STAR aligner.

**Supplemental Figure S3. Tree-way Venn diagram illustrating overlap of genes differentially expressed at differing stages of human plasma cell differentiation.** The largest changes in differential gene expression was identified between memory B cells (MBCs) and preplasmablasts (prePBs) stages.

**Supplemental Figure S4. Functional analysis of the differentially expressed genes between two consecutives stages of plasma cell differentiation.** Pathway enrichment analysis were performed using the Reactome R/Bioconductor package. The top 10 enriched pathways are shown for each transition step. (A) Upregulated genes; (B) Downregulated genes.

**Supplementary Figure S5. Temporal gene expression profile during human plasma cell differentiation**. Heatmap illustrating the gene expression profile of mRNAs showing transitions from A) low to high (one-step-up) or B) high to low (one-step-down) in two consecutive differentiation stages and C) two-step-up-down (up-down) or D) two-step-down-up (down-up) in the series of B to PC differentiation stages.

**Supplementary Figure S6.** Pathways enriched in A) one-step-down, B) two-step-up-down and in C) two-step-down-up genes.

**Supplementary Figure S7.** (A) High *BLM* expression in MMCs could predict for shorter overall survival. Patients of the Hovon cohort (N = 282) were ranked according to increasing BLM expression and a maximum difference in OS was obtained using the Maxstat R function. (B-C) High expression of BLM is associated with MM cell line resistance to Lenalidomide and SAHA HDACi. HMCLs response to drug treatment HMCLs were cultured in RPMI-1640 medium (Gibco, Thermo Fisher Scientific, Waltham, Massachusetts, USA) supplemented with fetal bovine serum (FBS, Eurobio, Les Ulis, France) (10%) and Interleukin 6 (IL6, Peprotech, Rocky Hill, New Jersey, USA) for XG cell lines. We evaluated the sensitivity of the cell lines to different drugs, including Bortezomib (Euromedex), Melphalan (HAC Pharma), Lenalidomide (Selleckchem), IKK2 inhibitor (AS602868), SAHA (Suberanilohydroxamic acid, HDACi, Selleckchem), and Dexamethasone. For a given drug, HMCLs were treated with different concentrations. The IC50was determined at day 4 using the CellTiter-Glo assay (Promega, Madison, Wisconsin, USA) as described^1^. The data represent the mean ± standard deviation of three independent experiments that were carried out on sextuplet culture wells. (D-E) ML216, induces a dose-dependent inhibition of cell growth in HMCL. HMCLs were cultured for 4 days in 96-well flat-bottom microtitre plates in RPMI 1640 medium, 10% fetal calf serum, 2 ng/ml of interleukin six culture medium (control), and graded concentrations of ML216. At day 4 of culture, the viability was assessed by CellTiter-Glo Luminescent Cell Viability Assay. The IC50 (concentration responsible for 50% of the maximal inhibitory effect), was determined using GraphPad PRISM software. Data are meanvalues±s.d. of five experiments determined on sextuplet culture wells.

1. Vikova V, Jourdan M, Robert N, Requirand G, Boireau S, Bruyer A*, et al.* Comprehensive characterization of the mutational landscape in multiple myeloma cell lines reveals potential drivers and pathways associated with tumor progression and drug resistance. *Theranostics* 2019; **9**(2)**:** 540-553.
